# An approach to heroin use disorder intervention within the South African context: A content analysis study

**DOI:** 10.1186/1747-597X-5-13

**Published:** 2010-06-23

**Authors:** Monika ML dos Santos, Solomon T Rataemane, David Fourie, Bruce Trathen

**Affiliations:** 1 Foundation for Professional Development, Pretoria, South Africa; 2 Department of Psychiatry, University of Limpopo, South Africa; 3 South African National Council on Alcoholism and Drug Dependence, Cape Town, South Africa; 4 Consultant Addiction Psychiatrist, sub-Saharan Reduction Network (SAHRN), South Africa & UK

## Abstract

**Background:**

The field of heroin use disorder intervention has been in transition in South Africa since the outbreak of the heroin epidemic. Yet despite growing evidence of an association between heroin users' use of supplementary intervention services and intervention outcomes, heroin use disorder intervention programmes in South Africa generally fail to meet international research-based intervention standards.

**Methods:**

Semi-structured interviews with ten heroin use disorder specialists were conducted and the interviews were subjected to content analysis.

**Results and Discussion:**

In terms of theory and practice, findings of the study suggest that the field of heroin use disorder intervention in South Africa remains fragmented and transitional. Specifically, limited strategic public health care polices that address the syndromes' complexities have been implemented within the South Africa context.

**Conclusions:**

Although many interventions and procedures have begun to be integrated routinely into heroin use disorder clinical practice within the South African context, comorbidity factors, such as psychiatric illness and HIV/AIDS, need to be more cogently addressed. Pragmatic and evidence-based public health care policies designed to reduce the harmful consequences associated with heroin use still needs to be implemented in the South African context.

## Introduction

In the not too distant past, almost anyone in South Africa could open a drug rehabilitation centre, offer rehabilitation services and charge a fee for these services - regardless of his or her professional training and background. These facilities were able to fall outside the ambit of the Mental Health Act (Act 17 of 2002) and the Prevention and Treatment of Drug Dependency Act (Act 20 of 2002) by calling themselves 'care centres'. Such facilities were thus not regulated by the Department of Health (DoH) or the Department of Social Development (DoSD). Numerous unregistered examples of such centres still exist in South Africa, and various human rights violations have been reported [1]. This situation has also arisen due to the state closing down several long-established centres and reducing subsidies for organisations such as the South African National Council on Alcoholism and Drug Dependence (SANCA). In 2007 the *Minimum Norms and Standards for Inpatient Centres*, which explicate the criteria for the registration of residential substance use disorder rehabilitation centres in South Africa, was issued by the DoSD [2]. However, the manpower to monitor the standards set is likely to be deficit [1,3].

According to Weich, Perkel, Van Zyl, Rataemane and Naidoo (2008), medical practitioners in South Africa are increasingly confronted with requests to treat patients with heroin use disorders, but many do not possess the required skills to deal with these patients effectively [4]. The collaboration between mental health practitioners and indigenous healers also needs to be further explored as indigenous healers are providing a significant mental health service to certain sectors of the substance use disorder population. In a study by Robertson (2006) it is estimated that 70% of South Africans consult indigenous healers, who include diviners, herbalists, faith healers and traditional birth attendants, while 61% of psychiatric patients had consulted indigenous healers during a 12 month period [5]. Furthermore, some treatment procedures, such as detoxification and rehabilitation can be especially expensive, and a large disparity between the services of the private and public health and welfare sector prevails. Well co-ordinated public health care policies that address heroin use disorders need to be implemented within the South African context, so as to prevent the spread of heroin use that could cause havoc across a continent already plagued by other tragedies such as HIV/AIDS [6-9].

### Goals of the study

The study explored heroin use disorder intervention strategies as imparted by a group of South African heroin use disorder specialists. The following goals were formulated for the study:

* To obtain expert opinion from South African heroin use disorder specialists with regard to intervention strategies that best facilitate heroin use disorder recovery.

* To furnish a description of the effective and ineffective aspects of heroin use disorder intervention from the perspective of South African heroin use disorder specialists.

* To formulate context appropriate recommendations, based on established evidence-based best practice research and the outcomes of the study, for the advancement of heroin use disorder intervention programmes within the South African context.

## Method

Purposive sampling was undertaken and semi-structured individual interviews with 10 heroin use disorder specialists were conducted from April 2007 to November 2009, ranging from 24 to 42 minutes. Potential participants were recruited based on their reputation for being heroin use disorder specialists within the South African context; as all the authors have practised in the field for between 10-25 years, identifying prominent specialists was greatly facilitated. All specialist participants gave their consent to participate voluntarily in the study. The small sample size was deemed adequate due to the in-depth nature of the study [10]. The specialists were asked the following initiating question:

'What interventions, both psychosocial and pharmacological/medical, are in your opinion the most effective in treating heroin use disorders?'

A standardised plan of the interview was read out to each specialist participant, and they were assured of confidentiality and anonymity. Two professionals that were approached for participation in the study, a psychiatrist and clinical psychologist, were unable to participate in the study due to professional and personal pressures. Ethical approval for this study was granted by the Ethics Committee of the Psychology Department, University of South Africa, in February 2007.

A qualitative descriptive approach guided the theoretical framework of this study. Content analysis was employed to analyse the interview information, such methods have been shown to be particularly valuable in the development of public health care interventions. This technique of analysis was also considered to be especially relevant considering the scarcity of research within the heroin use disorder intervention field in South Africa. Axial and sub-axial coding was adopted in the analysis for the disaggregation of core themes. Transcribed interviews were read, subjected to content analysis by the first author, and discussed by all the authors in order to determine the usability of the material. The interview comprised separate meaning units. These units were determined by phrases or paragraphs that were able to stand on their own and generate meaning. The translation of interviews into such units involved using the participants' own language in order to best generate a true meaning of their language. Categories were established by removing the meaning units from the rest of the interview and applying phrases that would encompass several of these units at once in their totality. These categories were coded in order to identify the regularities. Categories that were clustered together became themes. The inductive categorising of themes within the interviews increased the inter-rater reliability of the study.

To authenticate interpretations, interview conclusions were taken back to all the specialist participants for possible enrichment and verification of interpretations [11,12].

Table 1 below summarises the study specialist participants and their demographic background particulars.

**Table 1:** Study specialist participants

Specialist	Profession	Years/Country of Specialisation	Organisation	Long-term voluntarily abstinent
**1**	Director of an established South African rehabilitation centre.	15 years. South Africa and the United Kingdom	NAADAC counsellor	Yes, dependent for 12 years, currently 15 years heroin abstinent

**2**	Psychiatrist	Over 25 years. Singapore, Hong Kong, Canada, Australia, the SADC region, Western and Central Africa, Thailand, United Kingdom, United States and South Africa	University - Head of Psychiatry Department, Central Drug Authority, South African Medical Research Council	No

**3**	Executive Director	18 years. South Africa and United Kingdom	Organisation that specialists in substance use disorder research, advocacy and policy formulation for South African state departments	Yes, 6 years dependent on heroin, currently 32 years heroin abstinent

**4**	Social Worker	16 years. South Africa	Gauteng Department of Social Development, NA/NARANON	No

**5**	Social Worker	10 years. South Africa and United Kingdom	State Hospital in South Africa, methadone out-patient facility in United Kingdom	No

**6**	Lay	18 years. South Africa	Tough *Love *South Africa - serves on a prominent national level	No

**7**	Medial Nurse	19 years. South Africa	South African National Council on Alcoholism and Drug Dependence (SANCA)	No

**8**	Medical Nurse	14 years. South Africa	South African National Council on Alcoholism and Drug Dependence (SANCA)	No

**9**	General Practitioner	8 years. South Africa	Psychiatric hospital - buprenorphine maintenance regimes specialisation	No

**10**	Clinical Psychologist/Anthropologist	15 years. South Africa	Psychiatric hospital, private practice	No

## Results

### The impact of the heroin use disorder epidemic in South Africa

All the specialist participants regarded the availability of heroin, and consequent heroin use disorder syndromes, to be a relatively new and concerning phenomenon in South Africa. The need to intervene on a national level with regard to drug dealers was highlighted as an imperative, especially taking into account the relatively early onset of the heroin problem in South Africa. The lack of manpower due to the dismantling of specialised police units such as the South African Narcotics Bureau (SANAB) was cited as a severe constraint in dealing with the heroin availability problem in South Africa. Corruption within law enforcement departments and amongst mental health and health professionals was also mentioned by the specialist participants. Due to the relatively new emergence of heroin use disorders in South Africa, a lack of specialised knowledge amongst professional people working within heroin use disorder field came to the fore; however, the specialist participants felt that this backlog of knowledge has improved markedly in recent years. Problems with regard to the monitoring of substance use disorder rehabilitation programmes were also cited as a barrier in the effective implementation of the National Drug Master Plan. One participant spoke of the stigmatisation of heroin use disorders in the traditional white Afrikaner Calvinistic community, which may, for example, have contributed to delays in the development of appropriate medical interventions.

'*South Africa has not kept up to speed with the rest of the world... it was seen as a non-white issue, and because of the Calvinistic culture of this country, it was seen as a moral issue*.'

### Intervention

Intervention was regarded by all the specialist participants to be a central aspect in recovery and deemed of high importance. The themes of intervention and recovery were regarded to be interchangeable. The need to initiate intervention as early as possible (namely, at the onset of the heroin use disorder) was cited as vital. Also, the need to thoroughly assess clients was thought to be pivotal in terms of planning the most appropriate intervention, such as psychotherapeutic, outpatient and residential, pharmacological, legal and statutory input. All specialist participants were of the opinion that the length of residential intervention depended on the chronicity of the individuals' dependence on heroin. However, some specialist participants were of the opinion that all heroin use disorders should be regarded as severe regardless of length of use.

#### Components of intervention

The necessity for information and education within intervention, both in terms of facilitating cognitive restructuring and enhancing professional staffs' therapeutic skills, was thought to be of significance in the study. The enhancement and improvement of therapeutic skills, professional qualifications, maintenance of professional boundaries as well as the incorporation of indigenous healers within the therapeutic intervention was noted. The specialist participants also emphasised that heroin use disorders need to be seen as primary health conditions (as opposed to solely social and moral problems). The need for education and accreditation within the substance use disorder treatment field in South Africa was also viewed as an imperative. Therapeutic competencies such as Motivational Interviewing, as opposed to confrontation, were deemed as effective means of intervention. High level early intervention and long-term care were highlighted as important strategies in treating chronic heroin use disorders. The need for adequate resources, such as skilled medical staff, as well as equipment and medication in residential facilities, was cited as key in treating clients holistically. The monitoring of those presenting with heroin use disorders on an aftercare and outpatient basis was deemed important in securing long-term abstinence and eventual recovery. Lack of accommodation and facilities such as halfway houses was cited as a vast obstacle relating to the long-term treatment and care of heroin users. A number of specialist participants felt that few resources could be found to assist the extremely progressed cases of persons presenting with heroin use disorder syndromes. The need for an adequate time period to detoxify those in recovery was regarded as vital especially prior to continuing on an outpatient basis with therapeutic intervention. The use of alternative therapies in treatment intervention, such as relaxation and exercise, was also supported within the scope of this study. A further treatment intervention component that was reported to be influential in producing positive effects was the adoption of a holistic approach, in addressing the heroin use disorder syndrome, underlying problems, and problems caused by the using of heroin. The range of targets include behaviours, coping methods, physical and psychological problems, co-morbidity, practical problems, social and relationship difficulties, self-awareness, problematic behaviours, lifestyle, circles etc., rather than just the heroin use disorder syndrome. The specialist participants emphasised the need for protected specialised drug units away from temptations, especially for youth, during early recovery.

#### Therapeutic intervention

Some specialist participants held the sentiment that most rehabilitation centres in South Africa are not sufficiently successful in the treatment of heroin use disorders, and that the need for more therapeutic specialisation is necessary. Problems with regard to residential treatment accessibility were also mentioned, especially for people with average or low-incomes. One of the factors considered to be of most value was the continued use of post-treatment aftercare and counselling, and the importance of having a safe environment to return to if required. Residential detoxifications were also regarded to be medically safer compared to detoxifying on an outpatient basis. The need for counselling alongside pharmacological and medical intervention was regarded as an important component to intervention.

#### Community based/outpatient intervention

The cost benefits of outpatient programmes were highlighted by several specialist participants. The financial viability and accessibility of such programmes especially for advanced individuals presenting with heroin use disorders that have little recourse to financial resources, was regarded as a major advantage to such intervention. The opinion was held by a number of specialist participants that outpatient treatment, as opposed to residential care, is more realistic in that it facilitates recovering heroin users to deal with demands and triggers within their environment without succumbing to heroin.

'*Inpatient treatment is expensive, and doesn't test if the person can be out in the community without suddenly using the stuff; that is, can you control the craving within a community where there are a lot of triggers, knowing that there is support available*.'

The use of outpatient facilities that supply supervised pharmacotherapeutic interventions, such as the outpatient methadone maintenance programmes in the United Kingdom, despite the association with accidental death, was cited as being beneficial in assisting recovering heroin users adapt to their new life-styles while still being supported and monitored. One of the drawbacks of residential treatment, especially long-term residential treatment, was the possibility of inadvertently institutionalising individuals presenting with heroin use disorders and not facilitating their ability to cope in the outside world. The need to offer regular structured outpatient programmes, which enhance self-efficacy skills, was regarded to be of fundamental importance in the rendering of efficient services. Formulating a contract with clients, such as regular random urine testing, and the stipulation of possible consequences, was also highlighted as key in the rending of such services. Compliance to such programmes was found to be better with heroin users that had an active support system, employment etc. as opposed to those with minimal resources.

#### Pharmacological intervention

Substitute pharmacological intervention was generally supported by the specialist participants, especially those with a medical background. However, some specialist participants reflected that a significant number of persons in recovery maintain their abstinence with supportive therapeutic input only.

Two alternative modes of intervention were discussed by the specialist participants, namely no use of substitute pharmacology, implying the use of only symptomatic pharmacotherapy, the alternative being substitute pharmacotherapy such as methadone and buprenorphine. A number of the specialists felt that the pharmacotherapy intervention should not be of a long duration. The aspect of sleeping difficulties was noted as another important aspect that needed to be addressed pharmacologically in recovery as sleep deprivation is often regarded as a high risk factor for relapse.

Criticism against the use of pharmacotherapy was voiced mainly from specialist participants without medical training. The sentiments were shared by these specialist participants that long-term voluntarily abstinent heroin users who were not on any form of substitute maintenance programme, were more likely to remain completely abstinent from all forms of psychoactive substances. The use of opiate maintenance programmes was generally seen as a form of substitution, without addressing the underlying psychological and physiological factors. The negativity of prolonging the dependence syndrome was cited as a prominent criticism against pharmacological maintenance programmes. However, based on the outcome of this study, pharmacological intervention appears to continue to play an important role in heroin use disorder recovery as the phenomenon involves some degree of physical dependence.

### Incarceration

The lack of sufficient medical and rehabilitative initiative within prisons, especially those awaiting trial, was highlighted as a serious problem within the Correctional Services structure within South Africa. Heroin users who struggle with withdrawal symptoms seldom obtain necessary medical and pharmacological assistance. Furthermore, individuals with a heroin use disorder serving long-term sentences rarely obtain any form of specialised care for their syndrome. The incarceration of persons with heroin use disorders presenting with no co-morbidity or anti-social type personality traits was generally held in disdain by the specialist participants. There was general consensus amongst the specialist participants with regard to the establishment of community programmes, diversion programmes and rehabilitation instead of incarceration. The need to lobby government to be more proactive in their stance toward heroin use disorder rehabilitative initiatives was seen as a necessity.

### Harm reduction intervention

Harm reduction interventions such as needle exchange programmes, though contentious, were discussed at length by all the specialist participants. Two medical specialist participants, who are both internationally acclaimed substance use disorder intervention experts, stated that since the implementation of needle exchange programmes overseas (such as in Holland), a significant decrease in health-related consequences has failed to actualise. One specialist participant reiterated the fact that under international law (regulated by the International Narcotics Control Board), heroin remains an illicit substance, this factor adversely impacts on and makes the implementation of needle exchange programmes complex.

'*Harm reduction is a debatable issue. Unfortunately I have to quote the existing international law within the legislation, which is clearly regulated by the International Narcotics Control Board, and implemented by the UNODC - United Nations Office for Drugs and Crime. Heroin is an illicit substance, and it is listed as such*.'

All specialist participants were in agreement that needle exchange programmes are generally implemented with the intention of reducing harm associated with intravenous heroin use, such as the contraction of HIV/AIDS and hepatitis B, C and G. A number of specialist participants felt that needle exchange programmes might also maintain individuals presenting with heroin use disorders at a user level, extenuating their syndrome. The sentiment was held by some specialist participants that as mental health and health-care professionals, the goal of intervention should be to heal - and not to maintain heroin users' at the level of dependence. Another concern voiced by some of the specialist participants was that needle exchange programmes may further discourage heroin users from seeking rehabilitation intervention. Such programmes may also be promoting the use of heroin, sending mixed messages to impressionable individuals. A number of specialist participants also held the view that programmes such as needle exchange are too extremist. A few other specialist participants were of the opinion that it is immoral to condone and implement such programmes. One of the specialist participants mentioned that another problematic aspect with regard to needle exchange programmes, and so-called responsible use, arises with regard to progressively increasing tolerance levels and that inability to maintain dosages. The opinion of some specialist participants was that abstinence is not encouraged in such programmes, and that the heroin use disorder syndrome is perpetuated.

'*You're actually encouraging the addiction and you're not going to stop it like that because I don't think that one guy who's really severely addicted... you will not be able to maintain a dosage of that, that's my experience with addiction. I mean he will always want more*.'

Concern was also verbalised by some specialist participants that an intravenous users' family, and in particular children, might also be drawn into the heroin subculture due to exposure and by experiencing dualistic messages from their parents and other family members. Comparisons were made between that of intravenous heroin use and STDs in terms of advocating abstinence and implementing harm reduction approaches such as condom use. The comment was made by one specialist participant that once an individual is sexually active or dependent on heroin, for example, the likelihood of abstinence and behaviour change is not prevalent or always realistic. In such instances, harm reduction approaches may be the only manner in which risks may be reduced; be it through needle exchange programmes (regarding intravenous drug use) or condom use (regarding sexual risky behaviour). Another specialist participant verbalised that South Africa (as well as SADC and the African Union) is not at present in a position to support needle exchange programmes due to a lack of monitoring infrastructure and resources. Due to this factor, the specialist participant was of the opinion that stipulations of the United Narcotics Convention should still be adhered to within the South African context.

'*For substances that are as destructive as what heroin is, I think needle exchange provision of clean syringes and so on, is not something you can recommend in a country such as South Africa, in the SADC region, in the African Union countries, because we don't have the infrastructure to monitor, and we don't have the resources to provide these things*.'

Another factor mentioned by numerous specialist participants was that at present intravenous use in South Africa is still fairly low compared to that of other countries where needle exchange programmes have been implemented. However, these specialist participants commented that should the intravenous drug use level rise (which it is likely to), then needle exchange programmes in South Africa are likely to become necessary as part of strategic public health care interventions.

### Intervention key principles

The general measures of heroin use disorder intervention that emerged from the study are mostly long-term measures, aimed at bringing about long-term and fundamental changes. They are often collectively described as 'rehabilitation'. The following intervention principles emerged from the study:

* *Clarity of purpose *- Are the goals of the treatment clear and are both provider and recipient in tune regarding these goals? Are there specific outcomes (whether proximal or ultimate) being sought?

* *Appropriateness to the presenting condition *- Is there a fit between the person's current condition and what is proposed by them?

* *Assessment and care planning *- Has there been a thorough assessment to inform the care plan? Is this assessment based upon sound practice and knowledge and conducted skilfully? Is the care plan clear and well thought out?

* *Motivation for change *- Can the intervention cope with and respond intelligently to ambivalence, fluctuating motivation and varying degrees of realism? Does it meet the client where he or she is at? Is the client's choice understood and taken into account?

* *Preparation *- Is the client thoroughly prepared for the intervention to which he or she is referred? This applies to initial treatment or aftercare.

* *Supportive evidence and best practice *- Is evidence available to justify using the intervention and to indicate that it offers a better chance of success for the presenting condition than others? Has it been properly applied in practice? If there is a shortage of evidence, what is the rationale for employing the intervention? Does this make sense and is it testable? Qualitative and quantitative research may be able to make an important contribution here.

* *The whole person *- Are physical, psychological, social, cultural and spiritual factors taken fully into account?

* Families - Where appropriate and depending on circumstances, engaging with families and significant others at the earliest opportunity.

* *Responsiveness *- Is there an ability to adapt to changing circumstances?

* *Workforce *- Do its members possess the right knowledge and skill to be of real help and can they apply them effectively? Do they have adequate self-knowledge? Can they combine professionalism and vocationalism? Are they expertly led, supervised and managed? Is there a commitment to continuing professional development by both the employee and employer?

* *Harm reduction *- Are pragmatic and evidence-based public health care policies designed to reduce the harmful consequences associated with heroin use and HIV/AIDS implemented?

* *HIV testing *- Are HIV testing and treatment services made available in places accessed by vulnerable people? Fear of stigma and discrimination often keep injecting heroin users away from public health facilities.

* *Environment and culture *- Is it conducive to helping people to effect change? Is it attractive and does it encourage engagement? Does it signal care and invite confidence and trust? Is it structured, containing and safe? Is it positive and optimistic? Does it situate the client's welfare at the centre of its business? Does it help promote a recovery culture as opposed to a using one?

* *Scaffolding and recovery resources *- Individual interventions function better and last longer if the client is scaffolded by a support system to sustain motivation and is helped to develop personal resources so as to sustain treatment gains.

* *Organisation *- Is the organisation or service well run? Is it able to cope with the powerful projections associated with a distressed client group and avoid the associated pitfalls? Does it state a very clear ethical code which ensures that professional boundaries are maintained?

* *Quality control *- Are there active systems for auditing and monitoring processes and gaining client feedback?

* *The system *- Is the system for arranging and funding treatment, and in which treatment operates, coherent and therefore one that promotes effectiveness or undermines it? Are the pathways clearly defined and free from obstacles?

## Discussion

Findings of the study suggest that interventions should include a mix of approaches, both modern and indigenous, including assessment and diagnosis, pharmacological intervention, self-help intervention, outpatient, diversion and restorative justice approaches, residential care, and harm reduction tactics. However, as corroborated in the study of Odjide (2006), poor funding, insufficient skilled health personnel, poor laboratory facilities, inadequate treatment facilities, and lack of political will are some of the impediments to controlling heroin use in South Africa, as well as on the African continent in general [13].

The outcomes of the study suggest the importance of matching persons presenting with heroin use disorders to intervention. Assessment may identify the nature and severity of the heroin-related problem, to understand why it arose, to assess its consequences and to establish the strengths and weakness of the client and his or her situation; this finding is also mirrored in the studies of Dos Santos and Van Staden (2008), Terry (2003) and McIntosh and Mckeganey (2002) [14-16].

Once assessment is completed, the crucial question of how to help a heroin user with their problem has to be answered. The study findings suggest that the immediate response often needs to be pharmacological, although this is sometimes only a short-term measure and can only be regarded as one component of the total intervention response. A variety of counselling therapeutic intervention options were identified by the specialist participants: some are directed at the underlying causes which may have initiated heroin taking and/or contributing to its continued use, while others help to resolve the problems associated with or the consequences of heroin taking. Others dealt more with the heroin-taking behaviour itself; these are aimed at reducing or stopping heroin taking regardless of other problems or circumstances. This mix of approaches is further supported in the studies of Coombs (2004) and Rotgers, Morgenstern and Walters (2003) [17,18]. Findings of the study also indicate that not all interventions are suitable for every person presenting with a heroin use disorder, nor are they mutually exclusive. Intervention plans need to be drawn up thoughtfully, according to the needs of the individual. Single interventions or a mix of interventions, or components of different interventions, including indigenous healing practice, can be effective. Agrawal (1995) affirms that, specifically within the African context, it makes sense to talk about multiple domains and types of knowledge and intervention [19].

Study findings highlight that different treatment settings may be appropriate for different individuals in recovery. Residential hospital care may be appropriate for those with coexisting acute medical or severe psychiatric problems. Long-term residential care in a non-medical setting (such as halfway houses) may be most appropriate for those in recovery who are socially unstable, but who do not suffer from co-existing acute medical or severe psychiatric problems. Outpatient care, apart from its cost efficiency, may be indicated for socially stable individuals without coexisting acute medical or severe psychiatric problems. The differences in problems and in the individuals with these problems must be taken into account before one can make an informed decision about what type of treatment intervention is likely to be most appropriate, findings of which are supported by the studies of Dos Santos and Van Staden (2008), Gossop (2003) and Gruber, Chutuape and Stitzer (2000) [14,20,21].

Within the South Africa context, the collaboration between mental health practitioners and indigenous healers should perhaps also be promoted. The motivation for corroboration by the specialist participants is put forward as indigenous healers have been serving African communities since time immemorial, understand the belief system of their people, and enjoy a respected place in their society. By understanding and entering African religious and therapeutic expressions through its own language, important underlying, and possibly historic, commonalities and connections can be identified. The basis for variants and transformations can also be established more intelligibly and is supported in the studies by Mpofu (2003) and Janzen (1998) who explored indigenous healing practices in central and southern Africa [22,23]. Ultimately, more knowledge needs to be gained, widely shared, and debated, specifically about how indigenous healers practice, and what form of collaboration would be most appropriate.

The mixed study outcomes suggest that when illicit substance use can be prevented, every effort should be made to stop the problem before it starts. Where it cannot, creative means of dealing with the risks may have to be sought. The problem is that heroin use disorders are a kind of mental illness that do not manifest itself until it is too late. It sometimes makes sense to intervene at a political level. This is particularly true in cases where demand is not yet widespread - it may be in the best interest of society to make a collective decision not to allow certain substances to become popularised. As Leggett (2001) reiterates in his multiple studies undertaken within the South Africa context regarding the phenomenon; the question of any given society must be: is this strategy workable in the present circumstances [3]?

The specialist participants that favoured more regulated drug markets advocate a variety of harm-reduction techniques, such as allowing users to obtain their heroin or methadone from a government clinic or sponsoring needle-exchange programmes The state could participate in the market by providing the substance or the equipment under controlled conditions, thereby undercutting the illicit market, although international findings such as that of Adelekan and Stimson (1997) suggest mixed results [7]. Needle exchange programmes have perhaps the best-documented success rates of any harm reduction strategy. In 1998, the U.S. Surgeon General prepared a review of syringe exchange programs which concluded that, 'there is conclusive scientific evidence that (these programs), as part of a comprehensive HIV prevention strategy, are an effective public health intervention that reduces transmission of HIV and does not encourage the illicit use of drugs' [24]. The review noted an actual decrease in injection frequency among those attending the programmes. The study also identified these programmes as a unique opportunity to identify, refer and retain these high-risk individuals in local treatment programmess and other health services. However, the pragmatic harm reduction approach to heroin use disorders may not necessarily involve a comprehensive approach. When heroin use can be prevented, every effort should be made to stop the problem before it starts. The varied South African specialist participants' responses with regard to harm reduction strategies display the need for further debate, research and education on this subject. While it is unlikely that the South African government will be offering any free substances in the near future, condom distribution, for example, represents a very real concession to the view that endorsing abstinence may have its limitations.

### Practice, policy and future research implications

The results of this study indicate that heroin treatment programmes and early intervention are recognised as important responses that can indirectly have a great value in rehabilitation and HIV prevention programmes among those who use heroin. Existing interventions, however, lack the scale necessary to meet the growing needs of heroin users; likewise, the high cost of adequately scaled programmes for inpatient treatment may be a problematic. Outpatient treatment, support groups, diversion programmes as opposed to incarceration and community-based treatment and support could have a significant additional impact at a lower cost. Similarly to the findings of Affinnih (2002), the present study highlights the need for risk and harm reduction strategies to be translated and adapted to the specific conditions in the South African setting [8]. Learning from the best practice examples, as found in the study by Odejide (2006), of low-cost drug intervention strategies, community-based drug interventions, peer support groups and various harm reduction services like syringe and needle exchange programmes, opiate substitution treatment, community-based outreach and street-based services applied in other developing regions (e.g., in countries such as Iran, Pakistan, India, Cambodia) needs to be encouraged [13]. Non-governmental organisations and community-based organisations should be strongly encouraged to provide capacity building training to healthcare workers and law enforcement officers on working with heroin users. The use of innovative information technologies, in combination with traditional methods of knowledge-sharing and information exchanges, may be used to provide timely education. Making accreditation mandatory might also result in significant benefits for clients in treatment, especially those in residential care. HIV-related services - in particular, critical services such as voluntary counselling and testing - should be made known and accessible to those who use heroin and counselling services should be equipped to deal with heroin use-related issues. Counselling and testing services should be mobile and flexible in order to reach out to vulnerable populations in the communities.

Representative limitations may be evident in this study as different professional disciplines were interviewed across the spectrum of health and mental health, self-help and policy makers. However, the most easily identifiable persons were approached based on the authors' therapeutic, policy development and academic experiences within the substance use disorder intervention field, which ranged from 10-25 years, as well as on the objectives of the study. The sample size limitations also need to be noted. Ultimately however, the aim of this study was not to provide a basis for substantial generalisation, but rather to provide an explorative and descriptive account of heroin use disorder intervention from the view of a group of South African specialists. It is also important to note that the number of heroin use disorder specialists in South Africa was limited at the time that the study was conducted. Collecting data and information on the individual and programme levels needs to be further encouraged, as well as of the adoption of representative multilevel methods in future studies to examine the effects of various heroin use disorder interventions.

## Conclusion

Diagnosing, assessing and the management of interventions for those presenting with heroin use disorders can be quite complicated. The established HIV/AIDS epidemic in South Africa also compounds the situation. As confirmed in the South African studies of Myers (2005) and Myers and Parry (2002) treatment services, much of what is currently delivered as intervention within the South African context is based upon current best guesses of how to combine some science-based (for example, cognitive-behavioural therapy and pharmacotherapies) and self-help (12-step programmes) approaches into optimal intervention protocols [25,26]. It is no longer acceptable to simply do what feels right. Organisations and professionals who specialise in treating heroin use disorders are an accepted part of the health care delivery system. As in all other areas of health care, there is a rapidly increasing dependence on the development of scientific information within the South African context to shape and improve the future of the field. Within the past decade, psychiatrists, medical doctors, psychologists, social workers, family therapists, nurses and allied health professionals have all incorporated knowledge regarding the identification and treatment of heroin use disorders into categories of licensure and certification requirements. It is the ethical responsibility of the clinical practitioner in the heroin use disorder intervention field, as in other fields (such as cancer and heart disease), to stay informed with regards to new and more effective clinical procedures. Although many interventions and procedures have begun to be integrated routinely into clinical practice within the South African context, pragmatic and evidence-based public health care polices that are designed to reduce the harmful consequences associated with heroin use disorders need to be implemented.

## Competing interests

The authors declare that they have no competing interests.

## Authors' contributions

MMLDS drafted the original manuscript and undertook the data collection and analysis. The other authors advised and assisted in the interpretation of the data, technical quality of the paper and the development of policy recommendations based on the outcomes of the study. All authors; MMLDS, STR, DF and BT, have read and approved the final manuscript.
